# Beyond 4×: pathways to higher expansion factors in expansion microscopy

**DOI:** 10.1515/mim-2025-0040

**Published:** 2026-03-20

**Authors:** Josiah B. Passmore, Albert K. Serweta, Lukas C. Kapitein

**Affiliations:** Cell Biology, Neurobiology and Biophysics, Department of Biology, Faculty of Science, 84889 Utrecht University, Utrecht, The Netherlands; Visualise.bio B.V., Amsterdam, The Netherlands

**Keywords:** expansion microscopy, super-resolution, hydrogel chemistry

## Abstract

Expansion microscopy (ExM) enables nanoscale imaging with conventional light microscopes by physically magnifying biological specimens. While classical ExM achieves ∼4× linear expansion, advances in polymer chemistry and workflow design now routinely push expansion factors beyond 4×, thereby improving resolution while retaining compatibility with diverse labelling strategies. Here, we review current one-step and multi-step (iterative) high-expansion approaches, outlining how modifications to monomer composition yield highly swellable gels, and how iterative embedding can bypass the mechanical limits of any single polymer network. Finally, we discuss practical considerations and highlight how emerging strategies could maximise swelling capacity and simplify workflows. Together, these developments move the field toward scalable, reproducible, and broadly accessible nanoscale imaging.

## 1 Introduction

A push for higher resolution in light microscopy provides the ability to image biological structure at increasingly fine scales, revealing details that were previously accessible only by electron microscopy (EM). Expansion microscopy (ExM) [1] is a well-established protocol for the isotropic physical magnification of biological samples [2], [3], [4], [5]. By physically enlarging the specimen before imaging, ExM pushes light microscopy closer to the advantages provided by EM, with the potential to visualise organelles and proteins at near-EM resolution. This effect is compounded by the additional benefit of pan-labelling strategies [6], [7], which provide dense ultrastructural context alongside molecular specificity through fluorescence contrast, offering a complementary alternative to EM imaging. As a sample-preparation technique, ExM reduces reliance on specialised super-resolution microscopes – making high resolution imaging more affordable and accessible.

But to further push the limits of resolution, ExM can also be combined with a wide range of imaging modalities: when combined with optical super-resolution or computational fluctuation-based methods, theoretical effective resolutions can reach the single nanometer scale [8], [9]. Compatibility extends beyond microscopy – ExM can also enhance downstream omics techniques such as Fluorescence *In Situ* Hybridization (FISH), *in situ* sequencing, and proteomics by improving molecular accessibility and spatial separation [10], [11], [12].

Typical expansion factors in early ExM protocols reach approximately 4–5×, achieving ∼70 nm resolution on a confocal microscope. Because the effective resolution increase is expected to scale linearly with the expansion factor in both lateral and axial dimensions, pushing expansion factors higher yields direct benefits.

The original ExM hydrogel is built from three core components: (i) the nonionic monomer acrylamide (AA), providing a backbone for the polymer network and contributing to gel elasticity and mechanical stability, (ii) the ionic monomer sodium acrylate (SA), which incorporates into the polymer network and drives osmotic swelling through its negatively charged carboxylate groups, and (iii) the crosslinker *N*,*N*′-methylenebisacrylamide (BIS), linking the monomers together and stabilising the polymer network to preserve its shape. Following incubation in water, fixed negative charges on the polymer strands generate osmotic pressure that forces the network to hydrate and expand, while BIS crosslinks prevent dissolution, resulting in a stable, uniformly swollen gel.

So, what limits expansion factors to ∼4× in standard protocols? How can we achieve high-swelling gels and larger expansion factors, pushing the boundaries of resolution? The expansion limit of the original ExM formulations arises from the equilibrium between the osmotic pressure generated by SA, which drives swelling, and the elastic restoring force of the polymer network determined by BIS crosslinks. Increasing swelling would require lowering crosslinker density. However, as shown in the original ExM recipe [1] and in later attempts [13], [14], [15], simply decreasing the crosslinker concentration would mean compromising on mechanical integrity and isotropy.

In this review we will consider strategies for achieving >4× expansion, giving an overview of current approaches, while diving into the hydrogel chemistry that drives current recipe variations. We will consider single step approaches, which modify polymer chemistry to optimise ionic strength, as well as iterative approaches, which consist of multiple rounds of gel embedding and expansion. We will also consider practical considerations, and the advantages/trade-offs for these approaches. Finally, we will look at the possible future directions of the field.

## 2 Main body

### 2.1 One-step high-expansion strategies

Single-step expansion strategies attempt to overcome limitations by modifying polymer chemistry (Table 1; Figure 1). The primary goal is to increase swelling without sacrificing gel integrity, while also maintaining isotropy and protein retention. These modifications can differ in strategy, but aim is to optimise ionic monomer, nonionic monomer, and crosslinker concentrations to achieve stable low-density crosslinking. This optimisation comes from the fact that there is a trade-off: High concentration of crosslinkers leads to dense, structurally sound gels with low deformations, but this resistance comes with a difficulty to fully swell, leading to lower expansion factors. High concentration of ionic monomers leads to a higher swelling capability and possibly diffusion of proteins and antibodies increasing labelling efficiency, but comes with soft, watery gels prone to tearing and non-uniform expansion.

**Table 1:** Detailed overview of one-step high expansion strategies. Summary of published one-step high-expansion factor ExM methods, highlighting differences in approximate expansion factor (ExF), anchoring strategy, gel composition, and homogenisation strategy.

Name	Year	ExF	Anchoring	Monomers	Crosslinkers	Initiators	Homogenisation
20ExM	2024	∼20×	NAS	DMAA/SA	DMAA	KPS/TEMED	LysC/trypsin digestion, or SDS/DTT/heat protein disruption
3D-ExM	2024	∼12×	GA	DMAA/SA	DMAA	KPS/TEMED	ProK digestion/SDS protein disruption
BOOST	2025	∼10×	FA/AA	AA converted to ionic monomer via alkaline hydrolysis	BIS	APS/TEMED	SDS/heat protein disruption
GAMSI	2024	∼6×	AcX	AA/SA	BIS	APS/TEMED/4HT	Trypsin digestion
iPEX	2025	∼6×	NAS	DMAA/SMA	PAE	APS/TEMED	SDS/heat protein disruption
KA-ExM	2024	∼7×	MA-NHS	Acrylic acid/*in-situ* KA synthesis	BIS	KPS/TEMED	ProK digestion
Magnify	2023	∼11×	Methacrolein	DMAA/AA/SA	DMAA/BIS	APS/TEMED, or APS/TEMED/4HT	ProK digestion, or SDS/urea/heat protein disruption
NIFS	2022	∼9×	MA-NHS	AA/SA	EBIS	APS/TEMED	ProK digestion
PKA-ExM	2024	∼15×	MA-NHS	DMAA/KA	DMAA	KPS/TEMED	ProK digestion
ProteomEx	2022	∼8×	NAS	AA/SA	BIS	APS/TEMED/4HT	SDS/heat protein disruption
TREx	2022	∼10×	AcX	AA/SA	BIS	APS/TEMED, or APS/TEMED/4HT	ProK digestion, or SDS/heat protein disruption
X10	2018	∼10×	AcX	DMAA/SA	DMAA	KPS/TEMED	ProK digestion
Zoom	2019	∼8×	NAS, or FA/AA	AA converted to ionic monomer via alkaline hydrolysis	BIS	APS/TEMED, or V-50	SDS/heat protein disruption

**Figure 1:**
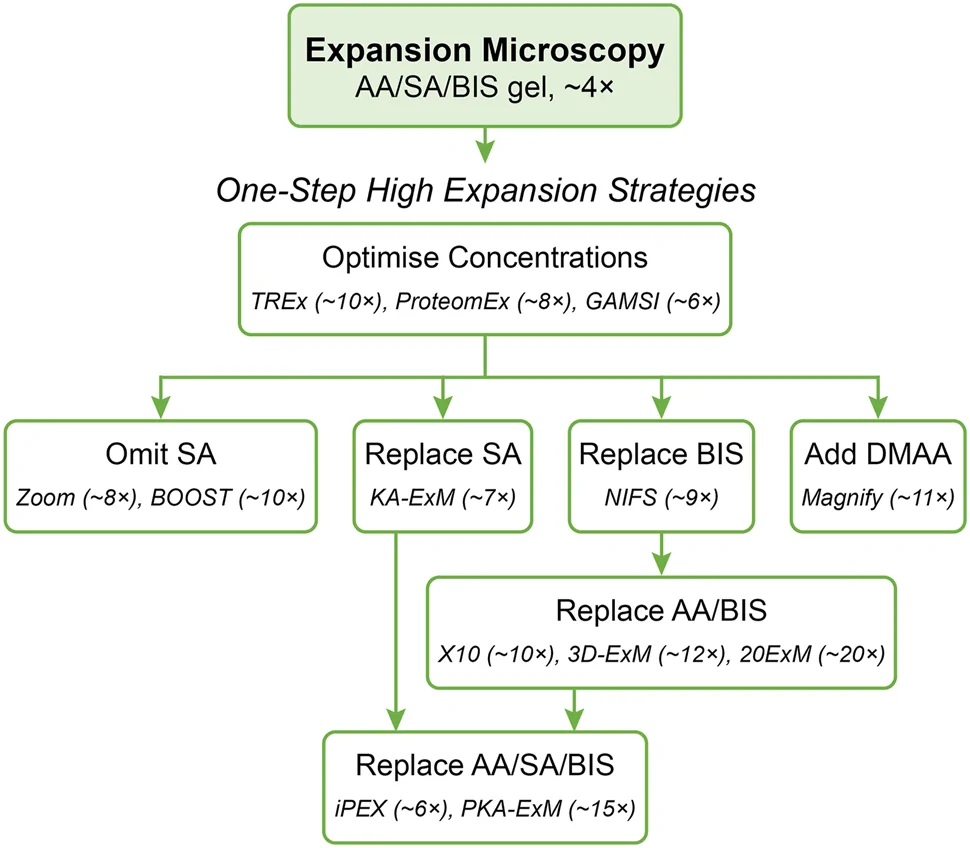
Overview of one-step high expansion strategies. Schematic flow chart summarising modifications to the original expansion microscopy protocol aimed at achieving expansion factors above 4× with a single gel. The first step (“Optimise Concentrations”) is common to all high-expansion variations, and subsequent branches illustrate alternative strategies modifying polymer composition. Abbreviations: AA, acrylamide; BIS, *N*,*N*′-methylenebisacrylamide; DMAA, *N*,*N*-dimethylacrylamide; SA, sodium acrylate.

#### 2.1.1 Variations using the core set of components

The original ExM recipe demonstrated high swelling by decreasing the BIS crosslinker concentration [1]. Exploring the parameter space within the core set of AA, SA, and BIS is one direction for high-swelling recipes. **TREx** [14] achieves **∼10×** expansion by pushing the classical chemistry toward extremely low crosslinking densities, while proportionally increasing the monomer content. Reducing BIS decreases the number of junction points in the network, while higher AA and SA concentrations provide enough polymer mass to maintain continuity during swelling. The optimised combination produced a high-swelling gel that can still polymerise efficiently and remain isotropic, unlocking high expansion factors without introducing alternative chemistries. A single-shot high expansion factor has proved valuable in proteomics, where proteins are retained for downstream mass spectrometry. Optimised AA/SA/BIS gels for high expansion factor omics are shown in **ProteomEx** [16] (**∼8×**) and **GAMSI** [17] (**∼6×**).

#### 2.1.2 Variations using alternative hydrogel chemistry

Departing from the three core components, several recipes utilise other replacements, with chemistry supporting higher expansion factors. In addition to replacing components, these recipes also optimise concentrations to support efficient crosslinking.

Replacing the ionic monomer SA for alternatives can lead to high-swelling, robust gels and allow for optimised ratios. In **KA-ExM** [18], SA is replaced with *in situ* formation of potassium acrylate (KA) from potassium hydroxide and acrylic acid, which reportedly increases mechanical stability of the gel. This allows further optimisation of the nonionic:ionic monomer ratio without suffering from loss of structure, leading to a robust gel with expansion factor **∼7×**. In **Zoom** [19], a dedicated ionic monomer is completely omitted. Nonionic monomer AA is converted to the ionic monomer via an alkaline hydrolysis step. The length of this hydrolysis step allows tuning of the expansion factor, demonstrated up to **∼8×**. **BOOST** [20] also utilises alkaline hydrolysis, but accelerates each step of the expansion workflow utilising microwave radiation. An **∼10×** expansion protocol was demonstrated in <2 h.

Alternative crosslinkers to BIS could present an opportunity for higher expansion factors, in particular longer length linkers. This was the focus of **NIFS** [15], which optimised gels with longer crosslinkers for isotropic swelling while maintaining gel stability. However, the longest crosslinkers often require additional polymerisation inhibitors to maintain stability, so NIFS ultimately settled on the slightly longer ethylene BIS (EBIS), forming a gel with ∼**9×** expansion factor.

*N*,*N*-dimethylacrylamide (DMAA)-based gels have been adopted as alternative options to AA/BIS. The self-crosslinking ability of DMAA means that it can function as the core monomer without the need to add a dedicated crosslinker. DMAA crosslinking and branching depends mainly on hydrogen extraction of its dimethyl groups. The resulting methylene radicals crosslink with each other, branch, or react with molecular oxygen if present. The latter renders DMAA polymers highly sensitive to oxygen and generally entails oxygen purging of gelation solutions and chambers. **X10** [21] provided the first demonstration of single-step **∼10×** expansion using DMAA. To reach full expansion oxygen was purged by bubbling the monomer solution with nitrogen. **3D-ExM** [22] achieved a slightly higher (**∼12×**) expansion factor without the need for purging the monomer solution of oxygen, by preventing air exposure during polymerisation using parafilm. In addition, glutaraldehyde (GA) was used as an alternative anchor to allow for higher swelling, likely by introducing longer, flexible linkers between biomolecules and the polymer network. **20ExM** [23] takes this concept further by not only deoxygenating the monomer solution with nitrogen dispersion, but also performing all steps in a nitrogen-filled glove bag. The efficient protection from oxygen resulted in a stable expansion gel of **∼20×**.

In addition to replacing AA/BIS with DMAA, some recipes have adapted DMAA gels further. Furthering the development of expansion proteomics, **iPEX** [24] presents a **∼6×** expanding DMAA-based gel, where sodium methacrylate is used in place of SA, and the crosslinker pentaerythritol allyl ether is added together with DMAA. Again, to prevent oxygen from interfering with polymerisation, gelation was performed in a nitrogen-gas-filled oven. These altogether result in enhanced rigidity, although at the cost of higher expansion factor. **PKA-ExM** [18] uses a higher-expanding DMAA-based hydrogel recipe but replaces SA with the oligomeric potassium polyacrylate (PKA) (in contrast to forming KA *in situ* as performed in KA-ExM). PKA products are superabsorbent polymers sensitive to ionic strength, and results in a single step **∼15×** expansion. To remove oxygen from the monomer solution, the buffer was subjected to cycles of vacuum degassing and nitrogen repressurising to replace any dissolved oxygen.

DMAA-based hydrogels without the requirement for rigorous degassing provide a more accessible way of achieving a higher and consistent expansion factor. **Magnify** [25] keeps the advantage of DMAA in forming lightly crosslinked networks via its inherent self-crosslinking reactivity, but retains a low concentration of AA and BIS. A high concentration of SA can then be added, resulting in a superabsorbent high-swelling (**∼11×**) gel with enhanced mechanical strength and compatibility, without the need for rigorous degassing.

#### 2.1.3 Outlook

Altogether, current demonstrations of one-step high expansion strategies have shown the capabilities of obtaining expansion factors far beyond 4×. These approaches are attractive to researchers because they maintain the straightforward workflow of standard ExM, while significantly improving resolution and remaining compatible with diverse labelling and imaging modalities. However, such high-swelling gels come with clear trade-offs. Gels become increasingly fragile and difficult to handle, prone to tearing or deformation, and slight inhomogeneities in polymerisation can introduce local anisotropy in a fully expanded gel. Ultimately, there appears to be a practical limit to how far a single polymer network can expand before mechanical instability, or nonuniform polymerisation/swelling dominate.

To push beyond this boundary while retaining sample integrity, iterative expansion strategies with multiple rounds of gel embedding and swelling have been developed, offering an alternative path to extreme physical magnification.

### 2.2 Multi-step (iterative) high-expansion strategies

Complementary to single-step high-expansion strategies, iterative expansion aims to progressively magnify the sample by building a new swellable polymer network inside the already expanded gel, allowing for another ‘round’ of expansion (Table 2; Figure 2). This then surpasses the mechanical limits of single gels while maintaining structural integrity and isotropy, avoiding the need for carefully balancing monomer/crosslinker composition. A neutral intermediate re-embedding (stabilising) gel is often needed, holding the original gel in an expanded state while transferring biomolecules and/or forming the next swellable gel.

**Table 2:** Detailed overview of multi-step (iterative) high expansion strategies. Summary of published iterative ExM methods, highlighting differences in approximate expansion factor (ExF), the number of rounds needed to reach that expansion factor, the anchoring strategy, composition of gels, and the handover and cleavage strategies.

Name	Year	ExF (rounds)	Anchoring	First swellable gel(s)	Homogenisation	Stabilising gel(s)	Final gel	Handover strategy	Cleaving strategy
Cyc-ExM	2022 (preprint)	∼20× (10)	FA/AA	AA/FA, V-50 (all rounds)	SDS/heat protein disruption	–	AA/FA, V-50	Direct transfer by AA/FA in gel	Heat
eMAP	2021	∼10× (4)	Direct retention	AA/SA/BIS, VA-044	SDS protein disruption	–	AA/SA/BIS, VA-044	No transfer (original gel retained)	No gel cleavage
ExR	2022	∼20× (2)	FA/AA	AA/SA/BIS, APS/TEMED/4HT	SDS/heat protein disruption	AA/BIS, APS/TEMED	AA/SA/BIS, APS/TEMED	No transfer (original gel retained)	No gel cleavage
hp-iExM	2017	∼16× (2)	Acrydite-modified oligos	AA/SA/DHEBA, APS/TEMED/4HT	ProK digestion	AA/DHEBA, APS/TEMED	AA/BIS, APS/TEMED	Hybridise new oligos	NaOH incubation
iExCel	2020	∼20× (2)	Acrydite-modified oligos	AA/SA/DATD, APS/TEMED/4HT	ProK digestion	AA/DATD, APS/TEMED	AA/SA/BIS, APS/TEMED/4HT	Hybridise new oligos	NaIO_4_ incubation
iExM (2-step)	2017	∼20× (2)	Acrydite-modified oligos	AA/SA/DHEBA, APS/TEMED/4HT	ProK digestion	AA/DHEBA, APS/TEMED	AA/SA/BIS, APS/TEMED	Hybridise new oligos	NaOH incubation
iExM (3-step)	2017	∼50× (3)	Acrydite-modified oligos	AA/SA/BAC, APS/TEMED/4HT (round 1) -> AA/SA/DHEBA, APS/TEMED/4HT (round 2)	ProK digestion	AA/BAC, APS/TEMED (round 1) -> AA/DHEBA, APS/TEMED (round 2)	AA/SA/BIS, APS/TEMED	Hybridise new oligos	TCEP incubation (round 1) -> NaOH incubation (round 2)
iterative umExM	2024	∼12× (2)	pGk13a, NAS	AA/SA/DATD, APS/TEMED/4HT	ProK digestion	AA/DATD, APS/TEMED	AA/SA/BIS, APS/TEMED/4HT	NAS incubation	NaIO_4_ incubation
iU-ExM (original)	2023	∼16× (2)	FA/AA	AA/SA/DHEBA, APS/TEMED	SDS/heat protein disruption	AA/DHEBA, APS/TEMED	AA/SA/BIS, APS/TEMED (U-ExM gel)	FA/AA incubation	NaOH incubation
iU-ExM (+TREx)	2023	∼25× (2)	FA/AA	AA/SA/DHEBA, APS/TEMED	SDS/heat protein disruption	AA/DHEBA, APS/TEMED	AA/SA/BIS, APS/TEMED (TREx gel)	FA/AA incubation	NaOH incubation
LICONN	2024	∼16× (2)	FA/AA, TGE/GMA	AA/SA/BIS, APS/TEMED/4HT	SDS/heat protein disruption	AA/BIS, APS/TEMED	AA/SA/BIS, APS/TEMED	No transfer (original gel retained)	No gel cleavage
pan-ExM	2020	∼16× (2)	FA/AA	AA/SA/DHEBA, APS/TEMED	SDS/heat protein disruption	AA/DHEBA, APS/TEMED	AA/SA/BIS, APS/TEMED	Direct transfer, FA/AA incubation (optional)	NaOH incubation
pan-ExM-t	2025	∼24× (2)	FA/AA	AA/SA/DHEBA, APS/TEMED	SDS/heat protein disruption	AA/DHEBA, APS/TEMED	AA/SA/BIS, APS/TEMED	Direct transfer	NaOH incubation
*re*-KA-ExM	2024	∼20× (2)	MA-NHS	Acrylic acid/*in-situ* KA synthesis/BIS, KPS/TEMED	ProK digestion	AA/BIS, APS/TEMED	AA/SA/BIS, APS/TEMED	No transfer (original gel retained)	No gel cleavage
*re*-PKA-ExM	2024	∼40× (2)	MA-NHS	DMAA/KA, KPS/TEMED	ProK digestion	AA/BIS, APS/TEMED	AA/SA/BIS, APS/TEMED	No transfer (original gel retained)	No gel cleavage
TG-based iExM	2021	∼16× (2)	Azide-modified oligos	Polyacrylate-azide TG monomer/PEG-SS-DBCO TG monomer	ProK digestion	AA/BAC, APS/TEMED	AA/SA/DATD, APS/TEMED/4HT	Hybridise new oligos	TCEP incubation

4HT, 4-hydroxy-TEMPO (4-hydroxy-2,2,6,6-tetramethylpiperidine 1-oxyl); AA, acrylamide; APS, ammonium persulfate; BAC, *N*,*N*′-cystaminebisacrylamide; BIS, *N*,*N*′-methylenebisacrylamide; DATD, *N*,*N*′-diallyl-tartardiamide; DHEBA, *N*,*N*′-(1,2-dihydroxyethylene)bisacrylamide; DMAA, *N*,*N*-dimethylacrylamide; ExF, expansion factor; FA, formaldehyde; GMA, glycidyl methacrylate; KA, potassium acrylate; KPS, potassium persulfate; MA-NHS, methacrylic acid *N*-hydroxysuccinimide ester; NaIO_4_, sodium periodate; NaOH, sodium hydroxide; NAS, *N*-acryloxysuccinimide; PEG, poly(ethylene glycol); pGk13a, palmitoyl-glycine-(d-lysine)13-azide; ProK, Proteinase K; SA, sodium acrylate; SDS, sodium dodecyl sulfate; SS-DBCO, disulfide-dibenzocyclooctyne; TCEP, tris(2-carboxyethyl)phosphine; TEMED, *N*,*N*,*N*′,*N*′-tetramethylethylenediamine; TG, tetra-gel; TGE, glycerol triglycidyl ether; VA-044, 2,2′-azobis[2-(2-imidazolin-2-yl)propane]dihydrochloride; V-50, 2,2′-azobis(2-methylpropionamidine)dihydrochloride.

**Figure 2:**
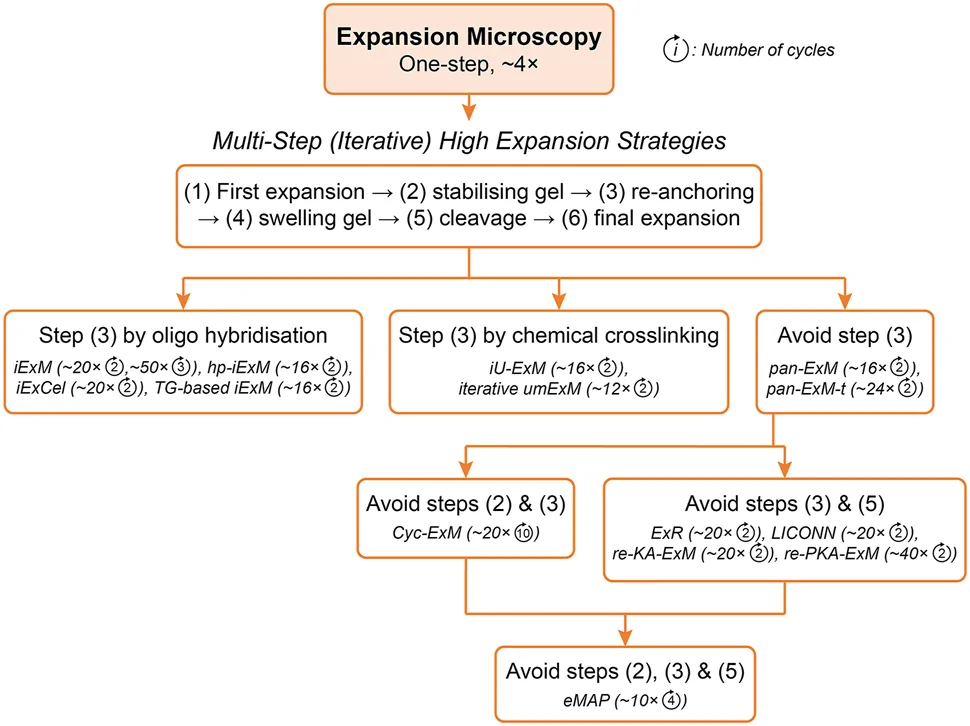
Overview of multi-step (iterative) high expansion strategies. Schematic flow chart summarising iterative approaches to achieve expansion factors above 4×. The core concept of iterative methods are to (1) perform a first expansion step with a swellable gel, (2) stabilise by re-embedding in a neutral, non-swelling gel, (3) perform a re-anchoring step to prepare biomolecules for gel handover, (4) embed into a second swellable gel, (5) cleave the original and stabilising gel, and (6) perform a final expansion. Subsequent branches illustrate various strategies with modifications on this core concept.

#### 2.2.1 Variations requiring cleavable crosslinkers

The first iterative ExM method, **iExM** [26], used the crosslinker *N*,*N*′-(1,2-dihydroxyethylene) bisacrylamide (DHEBA) as a replacement for BIS in the first swellable hydrogel, reaching approximately 4.5×. DHEBA is a cleavable crosslinker, which can be selectively cleaved by high pH conditions. Following the first expansion, the gel is re-embedded in a neutral, non-swellable stabilising gel, also cross-linked with DHEBA. As biomolecules were labelled with DNA oligos anchored to the network using acrydite groups, the stabilising gel ensures that the sample is held in the expanded state for hybridisation, applying a complementary oligonucleotide with a new acrydite group and a fluorophore. Following this, a second swellable gel is formed with BIS as a crosslinker, and the original and stabilising gel are dissolved by incubation in sodium hydroxide. The second gel can then undergo its own expansion of ∼4×, resulting in the final magnified sample of **∼20×**. A variation of the above, **hp-iExM**, sought to combine the dissolving step with hydrogel conversion via alkaline hydrolysis, omitting SA from the second gel. The expansion factors achieved were slightly lower, **∼16×**. To achieve a third round of expansion in iExM, orthogonal cleavage chemistry is required so that the first gel can be removed while still allowing the second to be dissolved at a later time. The first swellable gel was therefore crosslinked with *N*,*N*′-Cystaminebisacrylamide (BAC), containing a disulfide bond cleavable with a reducing agent such as tris(2-carboxyethyl)phosphine (TCEP). **∼50×** expansion could be demonstrated using three rounds of expansion.

**iExCel** [27] used an alternative cleavable crosslinker *N*,*N*′-diallyl-tartardiamide (DATD) in a recipe optimised for the expansion of *C. elegans*. DATD can be cleaved using sodium periodate without requiring high pH or reducing agents, potentially avoiding disruption of biomolecules. iExCel reached expansion factors of **∼20×**. For improved membrane labelling, iterative **umExM** [28] pre-incubates fixed samples with a dedicated membrane labelling probe and iteratively expands, also using a DATD-crosslinked cleavable gel with a final **∼12×** expansion factor.

When considering isotropic expansion, the free-radical chain-growth polymerisation of polyacrylate gels may contribute to structural defects. **TG (tetra-gel)-based iExM** [29] uses click-chemistry-based, non-radical terminal linking of tetrahedral monomers, that form highly homogenous gels with high structural preservation of biomolecules [30]. As the matrix is more structured, expansion factors are generally lower, however iterative expansion using tetragel monomers with a TCEP-cleavable disulfide-dibenzocyclooctyne (SS-DBCO) moiety in the first round, a BAC-based polyacrylate gel as the stabilising gel, and a DATD-crosslinked polyacrylate gel for the second round could again reach ∼**16×**.

**Pan-ExM** [7] built directly upon the principles of iExM using DHEBA-based gels, but aimed to improve molecular retention and labelling. First introduced in the tissue clearing method CLARITY [31] and later adopted for ExM in MAP [32], [33] and U-ExM [34], Pan-ExM utilised low concentrations of formaldehyde and acrylamide (FA/AA) to decorate biomolecules with anchors. FA and AA are small, highly soluble, and remain reactive in solution, unlike the bulkier, less soluble AcX. Combined with protein entanglement in the dense polymer network, FA/AA anchoring enabled superior proteome preservation across multiple expansion rounds. Final expansion factors reached ∼**16×**. An adjusted protocol for expansion of tissues, **pan-ExM-t** [35], yields higher expansion factors, **∼24×**, by optimising protein-protein crosslinking conditions.

Stringent epitope preservation and isotropic expansion of subcellular structure is the focus of iU-ExM [36] – a combination of U-ExM [34] and pan-ExM, again with DHEBA as cleavable linker – with an optimised homogenisation strategy and validation of isotropic nanoscopic resolution. As with pan-ExM, an expansion factor of ∼16× was demonstrated.

Avoiding a dedicated transfer step, removing the need of a stabilising gel completely would simplify the workflow significantly. Using a simple formaldehyde-crosslinked gel, **Cyc-ExM** [37] aims to reduce handover by simultaneously performing cleavage of FA crosslinks in the first gel, transfer of biomolecules to the next gel, and alkaline hydrolysis of AA for swelling in one high-temperature, high-pH denaturation step. However, the expansion factors per round are modest, requiring 10 consecutive rounds of embedding to reach **∼20×** expansion.

#### 2.2.2 Variations without original gel cleavage

Multi-step iterative ExM recipes that retain the original polymer network aim to preserve biomolecules and omit a dedicated transfer step from first to second gel. A high-swelling gel is formed in the space created by the original expanded gel.

**eMAP** [38], building on the original 4× MAP [32], [33] protocol, avoids covalently anchoring biomolecules to the hydrogel network by washing out formaldehyde and omitting a dedicated anchor, thus preserving epitopes that could be masked by chemical modification. A swellable but non-cleavable BIS-crosslinked hydrogel is used to retain biomolecules in the gel by physical entanglement. Expansion factors are deliberately limited per round to avoid requiring a stabilising gel, so that the impact of ionic strength on stability is minimised. Therefore, the final expansion factor is also limited, four rounds of embedding and a final expansion step results in a **∼10×** gel.

Expansion revealing (**ExR)** [39] also avoids gel cleaving, relying on further expansion of the first BIS-crosslinked gel by the second. A stabilising gel is needed to avoid loss of dimensional stability during formation of the second gel, but a transfer step is not required as the first is not cleaved, simplifying the workflow and ensuring minimal disturbance of biomolecules. A two-step iterative process results in a reported expansion factor up to ∼**20×**. In Multi-ExR [40], a final stabilisation step can be performed to achieve multiplexed (multi-round) staining in the high-expanded state. The same interpenetrating network-based non-cleaving approach using BIS-crosslinked gels is utilised in **LICONN** [41] for reconstruction of brain circuitry. An additional neutralisation of the original gel after polymerisation is used to ensure unreacted BIS is not crosslinked to the stabilising and final gel, and ∼**16×** expansion was observed.

#### 2.2.3 Combining iterative embedding with high-swelling recipes

A variation of iU-ExM using a TREx-like gel for the final swelling could reach ∼25× [36], but high variation within and between gels was noted, attributed to shrinkage of the first gel within the stabilising gel, amplified by the high-swelling TREx.

Building on the high-swelling potassium acrylate-based gels previously described, ***re*** **-KA-ExM** and ***re*** **-PKA-ExM** [18] use iterative approaches to reach even higher expansion factors in two-round embedding protocols – using the same philosophy as ExR and LICONN to retain the original gel and remove the need for biomolecule transfer. The *in-situ* KA formation approach reached maximum **∼20×**, and the PKA formulation reached the highest expansion factor shown for two-step iterative methods, at **∼40×**.

#### 2.2.4 Outlook

Iterative expansion strategies demonstrate that physical magnification of samples has the potential to far exceed the intrinsic mechanical limits of a single hydrogel network. By separating the total expansion into multiple rounds of swelling and embedding instead of relying on carefully tuned monomer ratios, these approaches have demonstrated expansion in the 10–50× range while preserving ultrastructural features and isotropy.

However, the strength of iterative expansion also introduces new trade-offs. Each embedding round adds workflow complexity, increases the risk of handling-induced deformation, requires cleavable chemistries or interpenetrating network formation, and biomolecule retention can vary depending on strategy. Stabilising gels are often required, maintaining structure during transfer.

Minimising the number of required transfer steps, more selective cleavage chemistries to allow an increasing number of rounds, swellable gels that maintain rigidity during embedding and combinations of high-swelling recipes with iterative expansion could streamline workflows, reduce distortion, and allow for maximum expansion factors.

### 2.3 Other parameters that influence the expansion factor

The chemical composition of the polymer network (Figure 3) has a strong effect on the final swelling capacity of the hydrogel. In addition, a variety of experimental conditions and sample-specific factors can significantly influence the achievable expansion. These include the kinetics of polymerisation, and the fixation, anchoring and homogenisation of the biological specimen itself, all of which interact to determine both the magnitude and uniformity of expansion.

**Figure 3:**
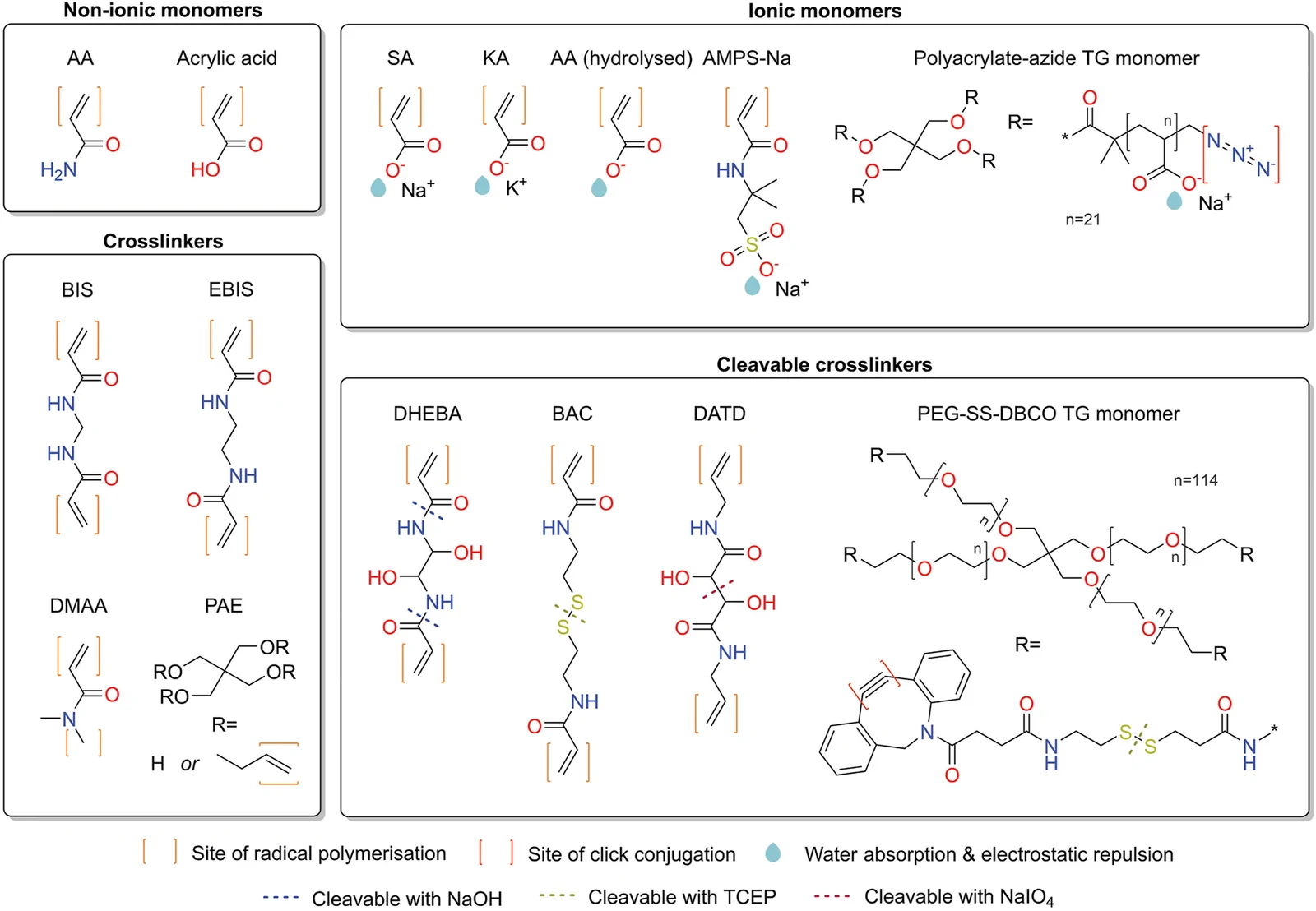
Structural comparison of monomers and crosslinkers used in expansion microscopy. Wireframe diagrams showing the chemical structures of monomers and crosslinkers commonly used in expansion microscopy. Abbreviations: AA, acrylamide; AMPS-Na, sodium 2-acrylamido-2-methylpropane sulfonate; BAC, *N*,*N*′-cystaminebisacrylamide; BIS, *N*,*N*′-methylenebisacrylamide; DATD, *N*,*N*′-diallyl-tartardiamide; DHEBA, *N*,*N*′-(1,2-dihydroxyethylene)bisacrylamide; DMAA, *N*,*N*-dimethylacrylamide; EBIS, *N*,*N*′-ethylenebisacrylamide; KA, potassium acrylate; NaIO_4_, sodium periodate; NaOH, sodium hydroxide; PAE, pentaerythritol allyl ether; PEG, poly(ethylene glycol); SA, sodium acrylate; SS-DBCO, disulfide-dibenzocyclooctyne; TCEP, tris(2-carboxyethyl)phosphine; TG, tetra-gel.

The kinetics of free-radical polymerisation strongly shape the final polymer network and thus the expansion factor. High initiator levels, high gelling temperatures, and fast-incorporating crosslinkers like bis-acrylamide produce rigid, heterogenous polymers with limited swelling capacity. Such conditions lead to formation of many short chains and rapid crosslinking early on during the gelation process [42]. In turn, lowering initiator levels and gelling temperatures has been shown to enhance swelling capabilities of AA/BIS gels. For instance, systematic titration of BIS in the rapidly polymerizing U-ExM protocol reached a plateau at around 11× expansion, but if the initiator content was decreased (from 5 to 1.5 mg/ml), U-ExM could reach a maximum expansion factor of 13× at low BIS concentrations without plateauing [14]. In AA/BIS gels crosslinking happens early on during the gelation process. However, in DMAA gels crosslinking occurs slowly and gradually. Upon incorporation into linear chains akin to AA or SA, DMAA requires further persulfate radical exposure to initiate branching and crosslinking [43]. This process likely happens over hours, gradually crosslinking the polymer and decreasing the expansion factor – as shown in 20ExM [23]. Further, ZOOM and BOOST protocols use polyacrylamide gels without any ionic component, very low initiator content, and apply heat-assisted, alkaline hydrolysis to convert amide groups on acrylamides into charged carboxyl groups [19], [20]. Such hydrolysed gels achieved 8–10× expansion and exhibited superior mechanical properties, compared to TREx [20]. This allows adjustment of the expansion factor with the length of the heat treatment using the kinetics of polymerisation, starting from a simple, polyacrylamide gel. These findings support the idea that a more controlled, slower gelation produces polymers with lower elastic restoring force that enhances swelling capacities.

The enhanced swelling and sturdiness of DMAA gels, relative to AA/BIS gels, can be explained by differences in chain length and crosslinker distribution. Crosslinker flexibility may further add to gel elasticity. In NIFS, where BIS is replaced with the longer, more flexible EBIS, the additional carbon atom in the linker region between the two acrylamide moieties roughly doubled the expansion factor of the original ExM recipe [15]. Similarly, the length between DMAA crosslinks can vary, likely enhancing gel elasticity. Another avenue for increased gel elasticity without the expense of stability is the use of reversible or self-healing crosslinkers. ExT [44] introduced sodium 2-acrylamido-2-methyl-1-propanesulfonic acid (AMPS-Na) as a crosslinker in expansion microscopy. AMPS-Na has ionic groups to drive swelling as well as sulfonic groups to create hydrogen bonds. The reversible nature of such hydrogen bonds adds stability without loss of elasticity.

While enhanced polymer elasticity benefits the expansion factor, decreased gel rigidity may compromise homogenous expansion of the biological structures within. Across various biological specimens, gels with higher expansion factors (and hence lower crosslinker densities), often exhibit incomplete expansion of denser structures when compared to blank hydrogels [36], [45]. This is particularly an issue with dense structures that are particularly difficult to homogenise, for instance large protein complexes [7], nuclei [7], [22], and challenging tissues [21], [23], [25], [27]. These artefacts can be minimised by increasing crosslinker density – at the cost of resolution – or applying stronger sample homogenisation to compensate for lower gel rigidity. In TREx, the lowest crosslinker density rendered gels too loose to uniformly expand thick brain sections, so aggressive sample homogenisation using both detergent-based heat treatment and protease digestion was utilised to achieve uniform expansion [14]. In X10ht-STED, autoclave-based heat denaturation allowed for ten-fold uniform expansion of cells and tissue sections [46]. In iU-ExM, a higher temperature denaturation step allowed more isotropic expansion of denser structures [36].

Another approach widely used in highly expanding gel recipes to prevent expansion factor mismatch between sample and gel is to reduce sample rigidity with gentler fixation methods. In organs and tissues, the addition of acrylamide to aldehyde-based fixatives offers two advantages. On the one hand, acrylamide reduces excessive crosslinking of biological structures during aldehyde fixation, resulting in softer samples. On the other hand, covalently attached acrylamides act as anchors for subsequent gelation. Such FA/AA fixation can allow for homogenous expansion of thick tissue sections with single-step recipes as well as iterative expansion protocols, solely based on heat denaturation [14], [35], [36], [46].

Yet another aspect that appears to affect the expansion factor is the physical linkage between gel and biological specimen. Numerous anchoring strategies have been shown to have an effect on the expansion factor, with multiple reports demonstrating incomplete expansion of structure when using AcX as the protein anchor, with increased concentrations limiting the expansion of cellular structures [22], [25]. Increased concentrations of fixative in combination with FA/AA anchoring has a detrimental effect on isotropic expansion factor in pan-ExM-t [35]. In the expansion of *C. elegans* in ExCel, lowering the concentration of LabelX results in 10 % improvement in the expansion of the animals without an effect on the quantity of detected RNA transcripts [27]. Multiple protocols explicitly terminate the anchoring step by washing with excess of neutral pH buffer before proceeding to gelation [16], [19], [24], [27], [46].

### 2.4 Determining the expansion factor

Reliably determining the expansion factor is crucial. When validating new recipes, checking robustness and isotropy of the gel, showing efficiency of homogenisation, and performing any quantitative assessments, being able to calibrate the expansion of the sample is critical. The most basic approach is to measure the macroscopic diameter of the expanded gel: dividing this value by the original gel diameter gives a rough estimate of the global expansion factor. However, variation between user measurements and non-isotropic expansion factors across the entire gel (arising from network heterogeneities from radical-induced polymerisation or bad-quality reagents, stretching or shearing from manual handling, or incomplete sample homogenisation) mean that a global measurement is unreliable [47].

The most reliable, yet labour-intensive way to determine the exact expansion factor of an imaged structure is to perform correlative pre- and post-expansion imaging of the same label. Registering the two images is often done to calculate deformation vector fields and the root-mean-square (RMS) error [1], but can also reveal the exact expansion factor, and photobleaching or photoactivating a known region [48], [49] can help to quantify expansion factors and distortions when performing pre-post correlation. Tools such as fluorescent coordinate systems that embed into the hydrogel together with the sample [47], 3D printed inserts that preserve shape and orientation [50], or obvious landmarks within the gel such as isolated glomeruli [51] improve pre-post correlation workflows.

Without pre-expansion imaging, ground truth markers provide an easier way to calibrate the gel locally. These intrinsic scale bars can be artificial with fixed dimensions, (such as protein-based fluorescent grids [47], DNA nanorulers [30], [52], nanopillars [53] or protein nanocages [54]), or biological with well-characterised average sizes (such as microtubules [55], nuclear pore complexes [56], or the membrane-associated periodic skeleton [45]). Calibration using biological averages over large fields of views using large heterogeneous structures (such as nucleus diameter) is not recommended without pre-post correlation, due to large variation and the known sources of anisotropy across gel regions. GelMap [47] allows such expansion factor calibration and deformation mapping across entire gels, without requiring pre-post correlation.

### 2.5 Future directions

Enhanced polymer engineering/rational design of expansion-optimised monomers would present a huge step forward in the field. Advances could include, (i) tuneable osmotic pressure or hydration properties of polymers to allow predictable or engineered network stiffness, (ii) radical-based chemistry with improved oxygen tolerance, (iii) non-radical based step-growth/click-polymerisation that reduce network defects and give predictable pore sizes, but do not compromise expansion factor, (iv) increased use of orthogonal cleavable linkers for many expansion rounds without chemical interference, (v) self-healing hydrogels that can dynamically repair network connectivity to possibly reduce anisotropy, but optimised for higher mechanical strength, and (vi) unified chemistries for anchoring and transferring between gels in iterative approaches, retaining proteins and reducing manual stabilisation/handover steps.

Combining high-swelling single-step recipes with iterative approaches has proven a powerful way to reach high expansion factors. However, typically a lower expansion factor, stable gel is used in the first instance, as any anisotropy or inhomogeneity in the first step will be amplified by the second. Solutions to increase the mechanical strength of high-swelling gels and also avoid the shrinkage of softer gels within stabilising gels will improve compatibility between the two approaches.

A key future direction for ExM is the development of more robust, universal and dense labelling strategies. Higher expansion factors place greater demands on labelling, due to dilution of fluorescence, amplification of linkage errors, and reduction of retention of pre-expansion antibodies. Post-expansion labelling can partially overcome these issues by accessing the more open polymer network but is limited by the density of retained biomolecules and potential epitope degradation. Future directions to address these challenges include the development of multifunctional linkers that simultaneously target biomolecules, carry reporter handles, and integrate efficiently into the polymer network [57], [58], [59], [60], and modified or recombinant antibodies specifically optimised for ExM to improve retention. Enhancing signal fidelity, labelling density, and versatility for both pre- and post-expansion approaches will support the next generation of high-resolution, high-swelling ExM workflows.

It is now routine to combine ExM with other imaging modalities to improve quality and resolution [9]. ExM together with optical hardware-based methods such as light-sheet microscopy [61], [62], [63], [64], [65], [66], [67], stimulated emission depletion (STED) [46], [68], [69], [70], [71] and structured illumination microscopy (SIM) [72], or with fluctuation-based computational super-resolution [25], [73], [74], [75] is accessible with few modifications to the ExM workflow. Combining ExM with single-molecule techniques [76], [77], [78], [79] adds an extra layer of complexity due to the use of buffers that may limit expansion factor, requiring low-expansion factor gels or embedding into stabilising gels. As increasingly stable high-swelling ExM protocols become available, compatibility with diverse imaging modalities will continue to grow, further extending the achievable resolution.

A key future direction for the field is the development of standardised, reproducible, and increasingly automated workflows, which will become especially critical as expansion factors continue to rise and in parallel ExM moves beyond research laboratories into clinical, diagnostics and pathology settings, where consistency and reliability is essential. As high-swelling gels magnify not only biological structures but also any variations in sample preparation/handling defects or anisotropic expansion, protocols pushing the limits of expansion are far less tolerant to deviation than classical 4× ExM. It is important to establish common benchmarks for measuring expansion factor, isotropy, and biomolecule retention, allowing results to be meaningfully compared across laboratories. Automated handling, from gel casting and handling to buffer exchange and imaging would minimise user-induced variability and improve throughput. As the field continues to optimise polymer chemistry, gel mechanical stability, sample homogeneity and labelling, optimisation becomes more demanding, and these robust standards and automation will be essential.

## 3 Conclusions

When aiming for higher expansion factors, current research has shown multiple pathways, each with clear compromises and trade-offs. Although clear trends emerge across one-step and iterative high-expansion chemistries, these patterns serve as guiding principles rather than design rules. Each formulation represents a carefully optimised balance of monomer composition, crosslinking density, and swelling pressures. Small changes can shift gel behaviour in unexpected ways, with loss of gel structural integrity, biomolecule retention, or expansion factor. As with earlier generations of ExM chemistries, the interplay between these parameters is highly nonlinear, demonstrating the need for experimental exploration alongside rational design. Ultimately, the most successful high-expansion workflow for a particular purpose emerges combines polymer chemistry with the structural, biochemical, and optical needs of the sample.
